# Glyphosate affects the larval development of honey bees depending on the susceptibility of colonies

**DOI:** 10.1371/journal.pone.0205074

**Published:** 2018-10-09

**Authors:** Diego E. Vázquez, Natalia Ilina, Eduardo A. Pagano, Jorge A. Zavala, Walter M. Farina

**Affiliations:** 1 Universidad de Buenos Aires, Facultad de Ciencias Exactas y Naturales, Departamento de Biodiversidad y Biología Experimental, Laboratorio de Insectos Sociales, Buenos Aires, Argentina; 2 CONICET-Universidad de Buenos Aires, Instituto de Fisiología, Biología Molecular y Neurociencias (IFIBYNE), Buenos Aires, Argentina; 3 Universidad de Buenos Aires, Facultad de Agronomía, Cátedra de Bioquímica, Buenos Aires, Argentina; 4 CONICET-Universidad de Buenos Aires, Instituto de Investigaciones en Biociencias Agrícolas y Ambientales, (INBA), Buenos Aires, Argentina; Montana State University Bozeman, UNITED STATES

## Abstract

As the main agricultural insect pollinator, the honey bee (*Apis mellifera*) is exposed to a number of agrochemicals, including glyphosate (GLY), the most widely used herbicide. Actually, GLY has been detected in honey and bee pollen baskets. However, its impact on the honey bee brood is poorly explored. Therefore, we assessed the effects of GLY on larval development under chronic exposure during *in vitro* rearing. Even though this procedure does not account for social compensatory mechanisms such as brood care by adult workers, it allows us to control the herbicide dose, homogenize nutrition and minimize environmental stress. Our results show that brood fed with food containing GLY traces (1.25–5.0 mg per litre of food) had a higher proportion of larvae with delayed moulting and reduced weight. Our assessment also indicates a non-monotonic dose-response and variability in the effects among colonies. Differences in genetic diversity could explain the variation in susceptibility to GLY. Accordingly, the transcription of immune/detoxifying genes in the guts of larvae exposed to GLY was variably regulated among the colonies studied. Consequently, under laboratory conditions, the response of honey bees to GLY indicates that it is a stressor that affects larval development depending on individual and colony susceptibility.

## Introduction

Pollination mediated by bees is an important agricultural service for food production and the maintenance of plant biodiversity [1]. The honey bee (*Apis mellifera* L.) is the main insect pollinator in agricultural settings [2]. However, the increasing disturbance of the agricultural ecosystem affects negatively honey bee health because of chronic adaptation to environmental challenges. Optimal homeostasis and immunity depend on a proper nutrition and a low allostatic load over time [3–5]. The decline of the honey bee population is considered to be a consequence of multiple factors, such as exposure to agrochemicals, pathogens, parasites, extreme climate conditions and bad beekeeping practices [6, 7]. An overload of individual or social compensatory mechanisms by concomitant stressors can lead to sublethal symptoms or increased mortality [8].

Since the late 1990s, there has been a rapid and steady expansion of cultivated areas using transgenic organisms in many countries [9, 10]. Glyphosate [N-(phosphonomethyl)glycine or GLY] is a low cost and broad-spectrum herbicide that became increasingly used in herbicide tolerant crops [11, 12]. Nowadays, GLY is the most widely applied agrochemical and has been detected not only in tissues of genetically modified crops (GMC) but also in traditional crops and resistant weeds [13–15]. Consequently, honey bee colonies located near crops are increasingly exposed to this herbicide [16–18]. As a result, GLY traces have been detected in as many as 70% of honey samples from countries where GMCs are permitted [19]. GLY has even been detected in samples of organic honey [19]. This indicates the accidental exposure to which bees are subject, presumably due to their wide foraging range [20, 21]. Although other pesticides have been detected in brood food, such as royal jelly, there is no data available that shows the presence of GLY in this beehive product [22].

Recently, sublethal adverse effects have been reported in adult worker honey bees fed with food containing GLY concentrations around 2.8 mg of acid equivalent (a.e.) L^-1^, the highest herbicide concentration recorded in agricultural surroundings [23–25]. These assays showed impaired associative learning and reduced sucrose sensitivity in young adult bees when reared in the laboratory under chronic GLY exposure [26, 27]. Forager honey bees exposed to acute GLY doses displayed weakened cognitive capacities needed to retrieve and integrate information for successful foraging [26, 28].

Previous studies have shown detrimental effects of GLY on the development and growth of a wide variety of animals, including arthropods [29–31]. Honey bees are holometabolous insects that have four moults that allow their growth during the larvae feeding period [32]. Each moult normally occurs approximately every 24 hours. During this period, larvae feed while nurse bees take care of them prior to sealing the cells for pupation (120 hours post-hatching) [33, 34]. Delayed larval development, i.e. when larvae have an unsuccessful moult on a given day and display an earlier stadium than that expected, has been shown in brood combs from beehives located in crop surroundings with high levels of pesticide contamination, e.g. neonicotinoids, pyrethroids and carbamates [35]. GLY was not monitored. Under semi-field conditions, colonies located close to resistant vegetation sprayed with 2.88 kg a.e. ha^-1^ of GLY presented traces of the herbicide of up to 1.3 mg a.e. kg^-1^ in honey and up to 629 mg a.e. kg^-1^ in bee pollen baskets. Furthermore, traces of GLY from 1.23 to 19.5 mg a.e. kg^-1^ were detected in brood samples [36]. Even though this study showed that larvae can receive food containing GLY, it did not find notable effects on their survival and development. However, the indirect administration of GLY to brood via the nursing of worker bees makes the exposure conditions among larvae complex and heterogeneous. Moreover, honey bees display variability among colonies in susceptibility to diseases [37, 38] and to pesticides [39, 40] due to differences in nutrition [3] and genetic diversity [41–43]. Both individual and social immunity determine the susceptibility of each bee and its colony.

In order to determine the effects of ingesting food contaminated with GLY on honey bee brood, we exposed larvae using a controlled direct administration. We carried out an *in vitro* rearing [44] with different GLY concentrations contained in the larval food, on the assumption of a worst case exposure scenario, assessing developmental effects in each larva during the moulting period when the brood cell was still unsealed (120 hours post-hatching) [32, 45]. We reared larvae *in vitro* from 6 different colonies in homogenised conditions and noted the effect of genetic diversity on the susceptibility to GLY of larvae without social immunity. Finally, we evaluated qualitative changes in the expression of genes linked to detoxification and health in the gut [46–49], that acts as the first barrier to xenobiotics, as parameters of brood health and responsiveness to GLY.

## Results

### Changes in larval development of honey bees exposed to GLY

In order to detect changes in honey bee larval development associated with chronic exposure to GLY (for details of exposure see S1 Table), we quantified survival and successful moulting during the larval feeding period (i.e. during the first 120 hours post-hatching, S1 Fig). Due to the possibility of variation among larvae from different source colonies in response to the herbicide, we sampled brood from 6 colonies (A-F) to rear them under *in vitro* conditions.

**Fig 1:**
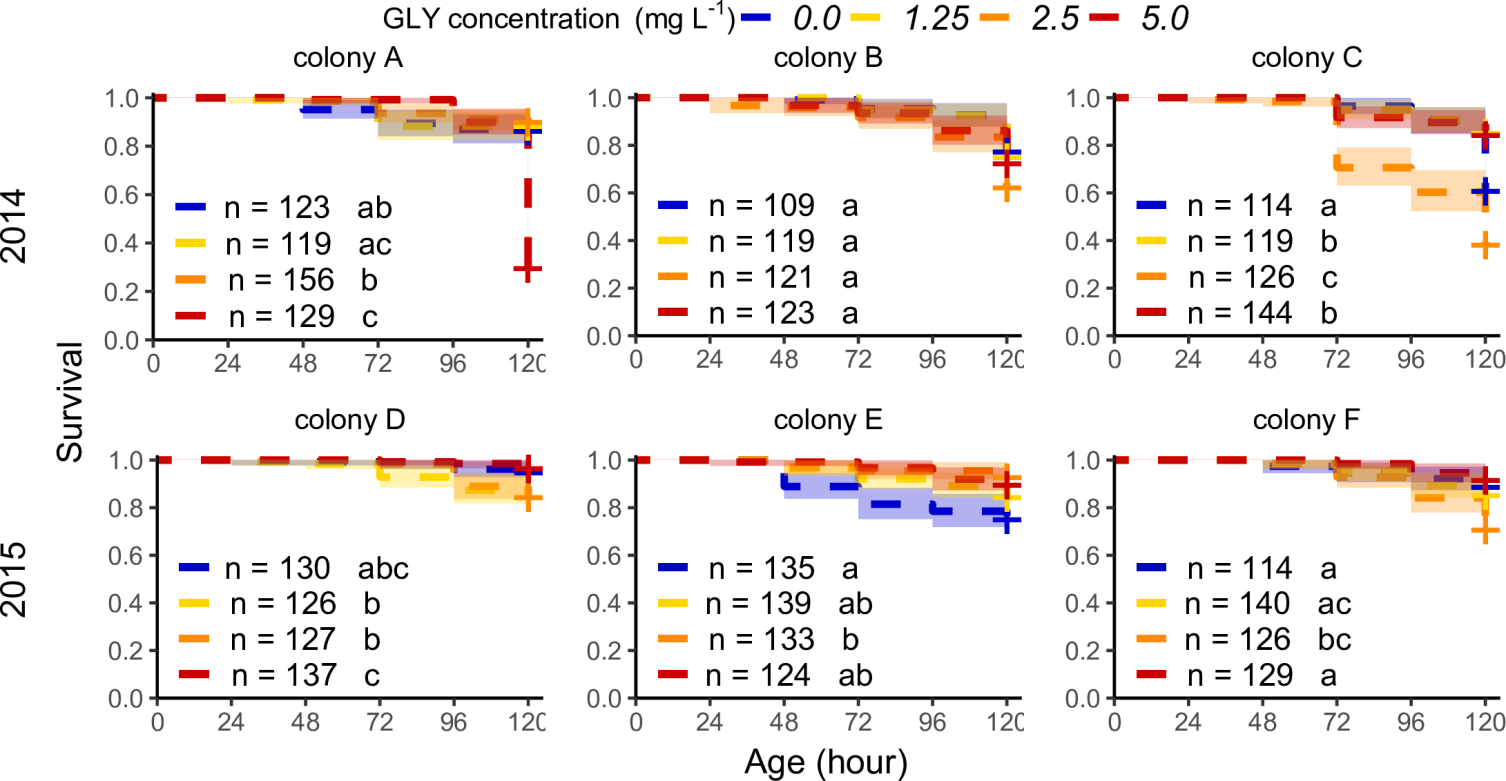
Larval survival under chronic exposure to glyphosate for different honey bee colonies. Proportion of larval survival during exposure (5 days post-hatching) to contaminated food with GLY (range of concentrations assessed: 1.25–5.0 mg per litre). Survival curves are plotted with their confidence interval (95%) for each treatment of larvae reared *in vitro* and for each individual colony (A-F). The number of assessed larvae is shown in the graph. Fitting of data to AFT model (survival prop. ~ [GLY] + colony + [GLY] × colony) followed by a Log-rank test for *post hoc* comparisons of simple effects. The curves are plotted with different colours per treatment: *in vitro* control in blue and a yellow-red gradient for increasing GLY concentration treatments. The + indicates time points with censoring data. Different letters indicate significant differences among treatments in each colony (S2 Table).

The mean age of death of all larvae in the control group was 92.9 ± 34 h, around the last moult before the fifth stadium. For larvae exposed to GLY, the mean age of death was similar, 92.2–106.6 h. Nevertheless, our results show effects on survival proportion with a significant interaction between the source colony of each larva and the GLY concentration administered (ATF model: survival prop. ~ [GLY] + colony + [GLY] × colony, χ^2^ (23) = 344.63, P < 0.001, N = 3062, *post-hoc* pairwise comparisons were performed with the log-rank test, S2 Table). Therefore, GLY affects larval survival with different patterns of dose-response among colonies (Fig 1). Colonies B, D and E did not show an adverse effect on survival, while the rest of the colonies showed a significantly lower survival after exposure to GLY (20–66% less of the baseline, Table 1). However, in colonies C and E, some GLY concentrations increased survival (22–39% more of the baseline, Table 1). We also found that the baseline of survival proportion was significantly different among colonies under *in vitro* rearing, when we compared the survival control curves (Table 1 and S3 Table).

**Table 1:** Variability among honey bee colonies in GLY effects.

Treatment	Effect (120 h)*	Year	2014			2015		
		Colony	A	B	C	D	E	F
Control	Baseline	Survival	0.86^ab^	0.77^bc^	0.61^c^	0.95^bc^	0.75^bc^	0.89^ab^
		Successful moulting	0.59^a^	0.56^a^	0.74^ab^	0.78^b^	0.61^ab^	0.54^a^
	Sample size		123	109	114	130	135	114
GLY exposure	Negative	LC (mg L^-1^) ^†^	5.0	NOEL	2.5	NOEL	NOEL	2.5
		Survival	0.29	-	0.38	-	-	0.71
		SLC (mg L^-1^) ^†^	2.5	NOEL	1.25–5.0	1.25–5.0	NOEL	2.5
		Successful moulting	0.35	-	0.00–0.48	0.21–0.52	-	0.30
	Positive	EC (mg L^-1^) ^†^	NOEL	NOEL	1.25, 5.0	NOEL	2.5	5.0
		Survival	-	-	0.85	-	0.92	-
		Successful moulting	-	-	-	-	-	0.75
	Range of sample size		119–156	119–123	119–144	126–137	124–139	126–140

**Fig 2:**
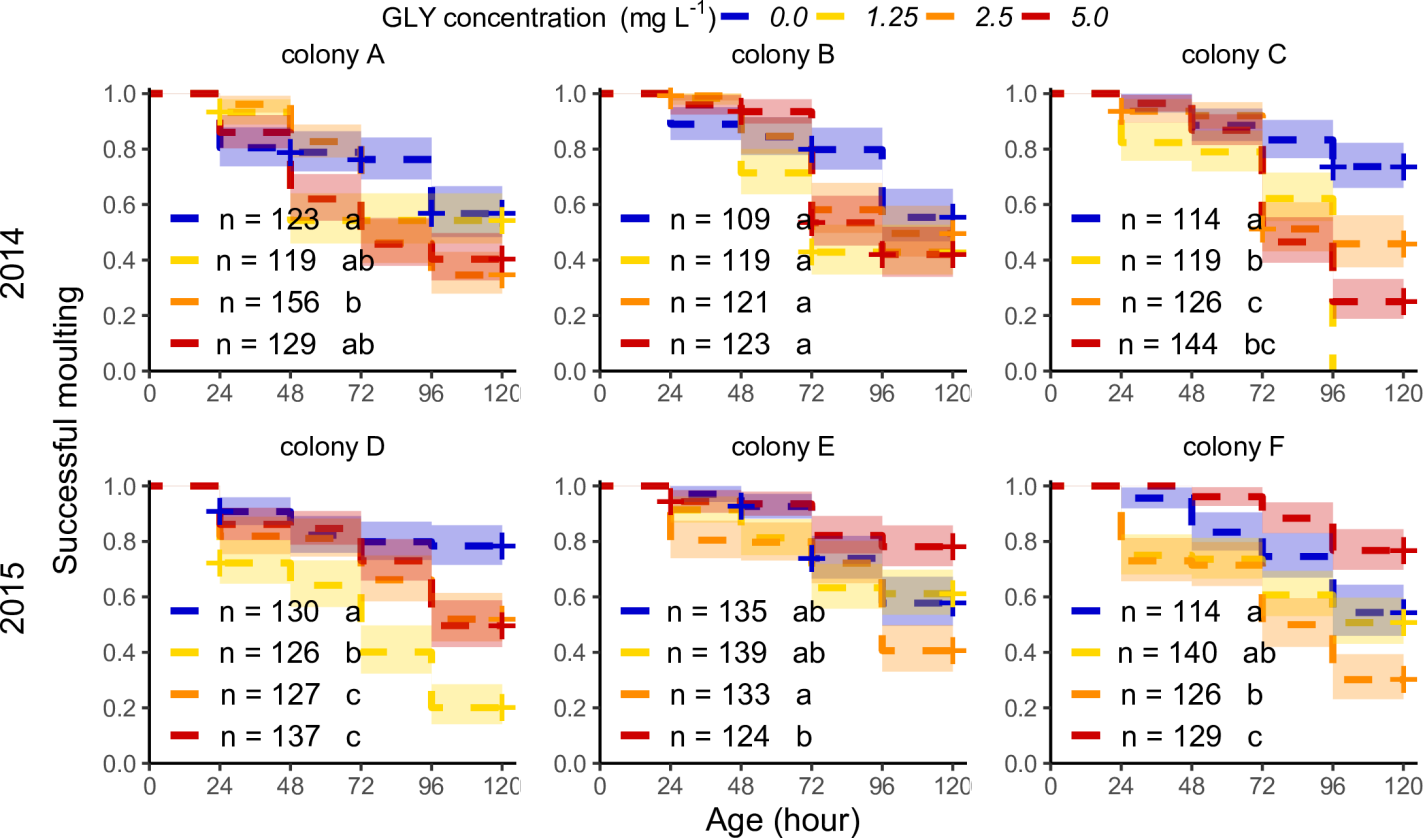
Larval moulting under chronic exposure to glyphosate for different honey bee colonies. Proportion of larvae without delay in moulting for each day during exposure (5 days post-hatching) to contaminated food with GLY (range of concentrations assessed: 1.25–5.0 mg per litre). Curves of successful moulting are plotted with their confidence interval (95%) for each treatment of larvae reared *in vitro* and for each individual colony (A-F). The number of assessed larvae for each treatment is shown in the graph. Fitting of data to AFT model (prop. of successful moulting ~ [GLY] + colony + [GLY] × colony) followed by a Log-rank test for *post hoc* comparisons of simple effects. The curves are plotted with different colours per treatment: blue for *in vitro* control and a yellow-red gradient for increasing GLY concentration. The + indicates time points with censoring data. Different letters indicate significant differences among treatments in each colony (S2 Table).

* Cumulative proportion of larvae with developmental effects during the exposure period to GLY (1.25, 2.5 and 5.0 mg of GLY per litre of food). The endpoints are death or delay in the moulting process (120 h post-hatching) measured in each larva. Fitting of data to AFT model (endpoint ~ [GLY] + colony + [GLY] × colony) followed by Log-rank test for *post hoc* comparisons of simple effects. The number of larvae assessed for each colony (A-F) is shown in the table. Baseline proportions with different letters are significantly different among colonies (S3 Table). This table is a resume of Figs 1 and 2.

† LC, lethal concentration; SLC, sublethal concentration; EC, effective concentration; NOEL, no observable effect level in range assessed. For each colony, only GLY concentrations that have significant statistical differences with its baseline are reported (S2 Table).

The developmental process can have sublethal adverse effects such as delayed moulting. The mean age of delay for larvae of the control group was 65.8 ± 35 h. This is consistent with the switch of diet within the hive from worker jelly to bee bread. The mean age of delay for larvae treated with GLY was similar, 60.7–68.2 h. Again, our results show a significant interaction between the source colony of each larva and the GLY concentration administered (ATF model: prop. of successful moulting ~ [GLY] + colony + [GLY] × colony. χ^2^ (23) = 409.2, P < 0.001, N = 3062, *post-hoc* pairwise comparisons were performed with the log-rank test, S2 Table). Therefore, GLY affects larval development with different patterns of dose-response among colonies (Fig 2). During *in vitro* rearing a number of unexposed larvae (22–46%) showed delay in the moulting process with variability among colonies (Table 1 and S3 Table). Under GLY exposure all the colonies, except for B and E, showed an increase in the proportion of larvae with delayed moulting (52–184% more of the baseline, Table 1). Only one concentration in colony F showed a reduced delay in development (46% less of the baseline, Table 1). The same proportion of larvae showed a double effect (i.e., delayed moulting followed by death) regardless of GLY concentration (33% for the control group and 26–38% for GLY exposed groups).

The effects on survival and development have shown a non-monotonic dose-response relationship with positive effects in certain cases. The lowest concentration of GLY induced early sublethal adverse effects in some colonies (C and D) after 72 h while the exposure concentrations of 2.5 and 5.0 mg L^-1^ induced sublethal and lethal effects in several colonies (A, C, D and F). This pattern could be a consequence of a response threshold in the detoxifying process. Accordingly, at the end of the exposure period assessed (120 h), the larvae exposed to 1.25 mg L^-1^ of GLY reached doses of around 100 ng of GLY per larva. Meanwhile, larvae exposed to 2.5 and 5 mg L^-1^ of GLY reached that dose within 72 h of exposure (S1 Table).

### Changes in growth after GLY exposure

As GLY exposure led mainly to developmental adverse effects, we wished to investigate what effect it might have on the growth of those 5-day old larvae that accepted the total amount of contaminated food (same nutrition and dose) without notable adverse symptoms. To assess this, we measured the head diameter and weight in larvae from three colonies (D-F). Given the differences in the rearing conditions and nutrition, we also compared the growth of larvae from the same cohort between rearing contexts (in-hive and *in vitro*, S2 Fig).

**Fig 3:**
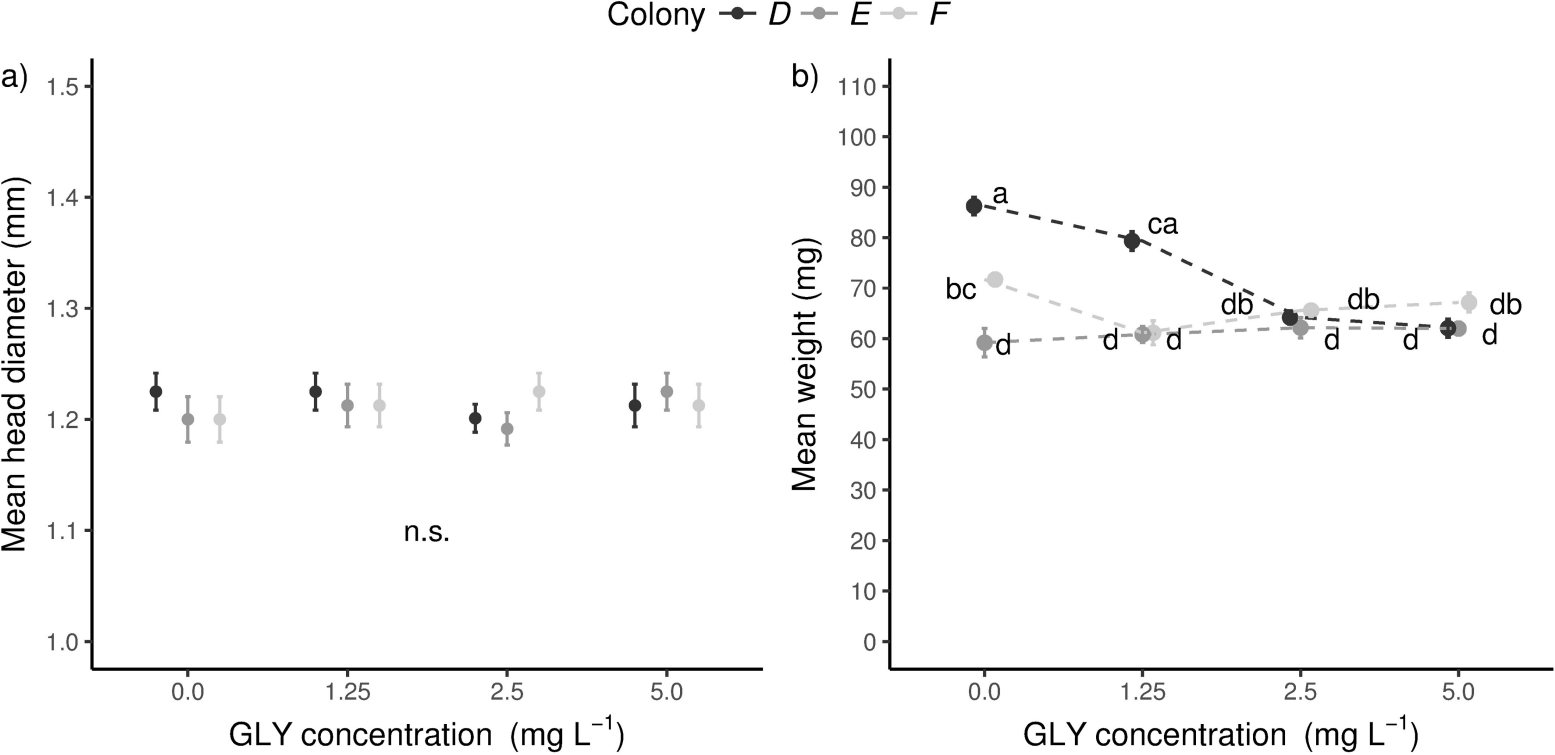
Effect of GLY exposure on growth. Larvae exposed *in vitro* to GLY (1.25–5.0 mg of GLY per litre of food) without conspicuous adverse symptoms in larval development were sampled at 5-day of age from three colonies (D, E and F). We measured in each larva their (a) head diameter (mm) and (b) weight (mg). The number of larvae measured was 10 for each concentration per colony. Kruskal-Wallis test followed by Nemenyi *post hoc* comparisons was carried out to analyse morphometric data to compare among groups (n.s., no significant differences). GLM (weight ~ [GLY] + colony + [GLY] × colony) followed by Tukey *post hoc* comparisons was carried out to analyse simple effects in weight data. Groups with different letters have significantly different means (S4 and S6 Tables).

Non-significant differences were detected in head diameter of sampled larvae among GLY concentrations (Kruskal Wallis test: head diameter ~ treatment. Treatment as a combination of [GLY] and colony, χ^2^ (11) = 19.74, P = 0.05; Nemenyi test, d.f. = (12, 108), no significant multiple *post hoc* comparisons, Fig 3A, S4 and S5 Tables). Therefore, these larvae were in the same growth stage associated with the fifth stadium as other studies reported previously [33,34]. Nevertheless, both source colony and GLY exposure explain significant differences in weight with a significant interaction (GLM model: weight ~ [GLY] + colony + [GLY] × colony. F(1,108) = 20.54, P < 0.001, N = 120, *post-hoc* pairwise comparisons were performed with the Tukey test, see Fig 3B, S6 and S7 Tables). Therefore, larvae exposed to GLY show a varying effect in growth among colonies. Colony E did not show an alteration in growth regardless of GLY concentration. Meanwhile, colony D showed a significant reduction of weight (around 27%) with a dose-dependent response in exposure to 2.5 and 5.0 mg L^-1^ of GLY. Furthermore, larvae from colony F showed a significantly lower weight (15%) for the lowest GLY concentration.

No larva with delayed moult ate all offered food in any of the colonies (A-F). Experimental food during the feeding period was a mixture of different nutrients, mainly from commercial royal jelly. The pH was slightly acid (4.94 ± 0.05). The pH did not change (4.9 ± 0.18) in food incubated during five days under similar conditions to those of larvae reared *in vitro*, which indicates the stability of some of its properties. Nevertheless, food became slightly more acid when GLY is added in concentrations of 2.5 and 5.0 mg L^-1^ (GLMM model: pH ~ [GLY] + (time|replicate). [GLY] term: F (3, 87) = 24, P < 0.001. Variance structure: 0.5% through days and 6% among replicates. S8 Table).

### Changes in gut gene expression of larvae exposed to GLY

*In vitro* rearing homogenizes environmental and nutritional factors among brood from different colonies. Therefore, the variability in effects is explained mainly by different genetic and/or epigenetic factors among brood, associated with the response to a xenobiotic. The first internal barrier to contaminated food is the gut. Hence, we performed an exploratory analysis of the expression profile of immune/detoxifying genes (Table 2). We sampled and dissected 5-day old larvae without adverse developmental effects from the colonies studied for morphological variables (D-F). We also analysed the expression profile of digestive enzymes and stress biomarkers genes (Table 2) to determine whether gut physiology had been disrupted as a result of GLY exposure. Finally, we compared gene expression between larvae reared in-hive and *in vitro* (S3 Fig).

**Table 2:** Specific primer pairs used to amplify each gene by RT-PCR.

Function	Target gene	Symbol	ID BEEBASE	Forward primer /
				Reverse primer
Detoxification	CYP6AS2	*CYP6AS2*	GB 49886	CTGAAAGATGGCGACCAATG
				GATCCCAAAAGCGCAACTAC
	CYP6AS3	*LOC726690*	GB 49887	GGGGTGTGCATGAATTCTCT
				GGCATATACAGCCTGGTGAA
	CYP6AS4	*LOC412209*	GB 49885	AGAGGTGGTCCTGTCGATTG
				CTTGGTCATGAACACGGTTG
	CYP6AS5	*CYP6AS5*	GB 49890	GGAACCATTATCACCGCATC
				CGAACACTTCTCTGCCCATT
	CYP6BD1	*LOC551560*	GB 47279	GTTGAAGCTGCCAATTCGAT
				ATGCTGCGAGAAAATGTCGT
	CYP9Q3	*LOC408453*	GB 43728	TTCAAGCTGATGACCGAGTG
				ATCTGTTGGTGCCCAACTTC
	Esterase FE4-like	*LOC409171*	GB 47299	GGCCCACTTCGATTTAAGGT
				GAAACGAGATGGGAACAACA
	Carboxylesterase	*LOC726134*	GB 47974	ACATTTCTGGGGCATCTCAC
				TGGGATGGAAGAGGCAATAG
	GstD1	*GstD1*	GB 50265	TTTCCGTCTGTGGGAAAGTC
				TCCCTGCCACATAGTTTTCC
Immunity	Abaecin	*LOC406144*	GB 406144	CACTACTCGCCACGATATGC
				CGGATTGAATGGTCCCTGAC
Stress marker	Hsp 70 Ab-like	*LOC410620*	GB 50609	GATTCGCAAAGGCAAGCTAC
				CCGCTGTTGACTTCACTTCA
	Hsp 70 cognate 3	*Hsc70-3*	GB 49117	CGATCAAAACCGCCTTACAC
				GGAATCGCTGACTTTTGAGC
Digestion	Cysteine proteinase	*LOC408851*	GB 44533	ACATTTGAGCAAGGGACAGC
				CGCGTATTGGCCTTCTACAT
	Cathepsin L1	*LOC552756*	GB 54331	AAAGATCAAGGCCATTGTGG
				ATCAATCCTCCATTGCATCC
	Alpha-glucosidase 2	*AGLU2*	GB 43248	AGAATGGCGAGATTTTGTGG
				AAATTGTTCTGGCGTGGAAG
	Alpha-amylase	*LOC406114*	GB 49854	ACGTCAGGTCGAAGCTTGTT
				TTCCGTTGTACTCCCGTTTC
Housekeeping	Actin	*Arp1*	GB 44311	TGCCAACACTGTCCTTTCTG
				GGAAGGTGGACAAAGAAGCA

On the one hand, expression levels of digestive enzymes and stressor biomarker genes were similar among colonies with low variability (in-hive: CVs 6–22%; *in vitro*: CVs 2–13%), suggesting that the *in vitro* rearing conditions do not disrupt gut physiology. Nevertheless, we did find high variability in the expression levels for some immune/detoxifying genes (in-hive: CVs 2–60%; *in vitro*: CVs 8–89%) among colonies in both contexts with a considerable trend to an increase in expression in the *in vitro* rearing.

**Fig 4:**
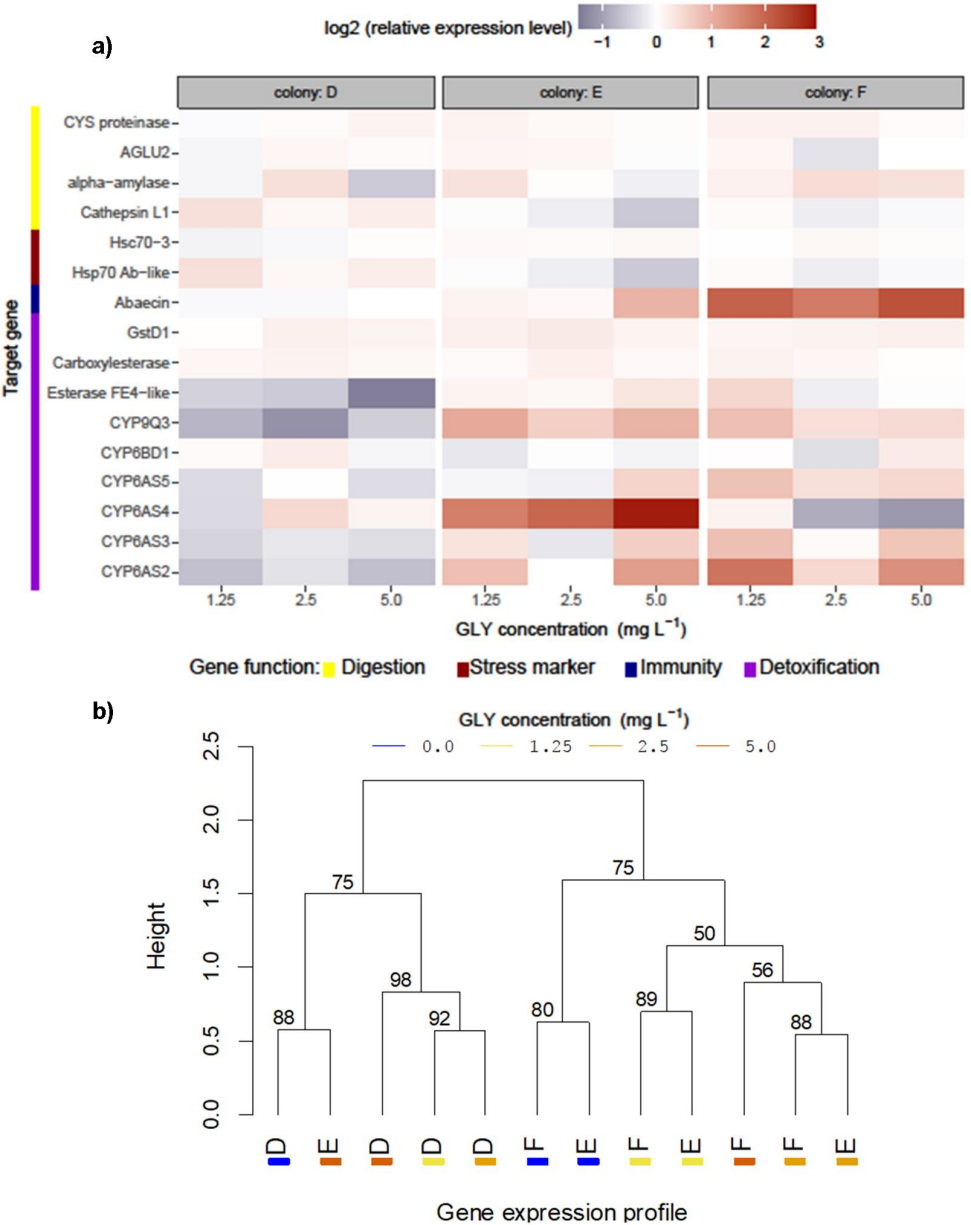
Variability among colonies in gene response to chronic GLY exposure. Measurement of gene expression levels in guts of 5-day-old dissected larvae (reared *in vitro*) from three colonies (D, E and F) exposed to different concentrations of GLY (1.25–5.0 mg of GLY per litre of food). A pool of 10 guts for each colony and treatment was assessed (12 samples). (a) The expression level of each gene in exposure to GLY was relativized to the baseline expression level of itself (in absence of the xenobiotic). A heatplot was plotted for each colony with its relative expression profile in each GLY concentration (logarithmic scale for relative gene expression). Colour scale: red for overexpression and blue for subexpression with respect to their baseline. (b) Dendrogram from hierarchical cluster analysis performed among samples with normalized gene expression (multiscale bootstrap resampling p-values for clustering in each edge).

On the other hand, we analysed the gut responsiveness of larvae exposed to GLY. Due to the different baselines of gene expression among colonies in control groups, we relativized each expression profile to the control sample to compare the responsiveness patterns (Fig 4A). Expression levels for digestive enzymes and stress biomarker genes seem similar between exposed and unexposed larvae. However, immune/detoxifying genes are regulated by GLY or its subproducts as a consequence of the exposure, with a differential response among colonies. Colony D showed a general downregulation of immune/detoxifying genes. Colonies E and F showed upregulation regardless of GLY concentration, especially in genes *CYP6AS2* and *CYP9Q3*, in both colonies. Colony F showed an upregulation of the immune gene *Abaecin* and a downregulation of *CYP6AS4* with a dose-dependent response opposite to that of colony E. The clustering method (Fig 4B) showed a similarity between expression baselines of colonies E and F when using their complete gene expression profiles. All samples from exposed larvae were grouped separately from control larvae (Fig 4B). In addition, the expression profiles of the samples exposed to the same concentrations of GLY in colonies E and F were grouped, indicating a similar response for both colonies. Thus, the variability in the regulation of immune/detoxifying genes could explain the variability in tolerance to GLY exposure.

## Discussion

The current *in vitro* assessment shows that GLY promotes changes in the proportion of brood with developmental alterations (i.e., changes in its prevalence), such as delayed moulting or even death, in honey bee larvae fed with contaminated food (1.25–5.0 mg of GLY per litre of food). This effect is observed mainly as a prolonged duration of early larval stadia and reduced growth. These negative effects for GLY treated larvae were found in the majority of the colonies studied (A, C, D and F) mostly with 2.5 mg a.e. L^-1^ of the active ingredient. However, the great variability among them with regards to susceptibility to GLY is remarkable. This varying response among colonies is explained by differences in the proportion of susceptible larvae in each colony (intra-colony genetic diversity) and by the different response thresholds. In four cases, the exposure concentrations induced positive effects in colonies C, E and F. Transcription of immune/detoxifying genes in larval gut was variably regulated among colonies after chronic exposure to GLY. Consequently, in absence of social immunity and other environmental factors, the inter-colony genetic diversity could explain the variability among colonies in developmental effects and susceptibility to GLY [42, 43, 50]. This could involve one or several genes in the mechanisms of compensation of the different genotypes resulted in tolerant phenotypes (asymptomatic bees). However, the tolerance to GLY does not imply its harmlessness, contributing to increase the allostatic load of a colony.

### Honey bee larvae response to GLY as a stressor

Studies in both vertebrates and invertebrates have shown cell cycle dysregulation and teratogenic effects with molecular links between GLY-based herbicides and the cellular rearrangement pathways that underlie animal development [51, 52]. Consistent with this, elevated levels of cell apoptosis in the gut epithelium of honey bee larvae were found after *in vitro* GLY exposure [53]. The transcription of some immune/detoxifying genes in the larval gut, such as the antibacterial protein gene *Abaecin* and some cytochrome P450 monooxygenases, is regulated by ingestion of GLY in our study. Similar results were found in a previous study [54]. Even though this effect was reported to be reversible, all these alterations in cellular physiology of gut are consistent with chemically induced stress [55] and a disrupted microbiota [56].

Adequate nutrition is essential to accomplish optimal development when facing exposure to stressors [3]. Young worker honey bees showed reduced intake of rearing food when it was contaminated with GLY [27]. However, larvae with delayed development do not eat all the food offered, regardless of whether they have been exposed to GLY or not. Specifically, the moulting process depends on growth rate, which is linked to feeding, that is homogenized in the *in vitro* assessment. As food is a constraint for the immunity of brood, its properties are important under these conditions. In this sense, the acidification of rearing food by GLY has unknown consequences. The triggering of stress compensatory mechanisms induces energy consumption which could disrupt the moulting process and could lead to death if larvae have low individual tolerance. Colonies D and F showed a reduction in larval growth even if larvae did not show delayed moulting. We do not know the causes of the reduction in feeding behaviour. However, malnutrition could lead to inefficient compensation to stress because of a trade-off between growth and detoxification [31].

We mainly detected adverse developmental effects *in vitro* for honey bee larvae with a non-monotonic dose-response, i.e. high responses at intermediate doses. Different hypotheses can explain this kind of pattern; metabolic effects, plurality of molecular targets, negative feedback regulation and multiple antagonistic mechanisms with different thresholds [57]. The dose-response depends on the duration of exposure and on the assimilation time of the substance. The relationship between the exposure concentration and the effective dose is not necessarily linear. Hence, positive effects can be explained by short term low dose exposure events that could enhance the health of individuals due to the regulation of immunity genes. Consequently, further studies are necessary to understand the mechanism of action of GLY ingested during larval development. Our assessment was focused on the larval stage, so subtle effects during pupation of tolerant larvae with or without reduced growth remain unknown.

### Implications of *in vitro* exposure conditions

The presence of GLY does not seem to modify the acceptance of the rearing food by neither tolerant larvae nor by forager honey bees [26]. This response was observed even though GLY slightly acidified the diet. This allows us to calculate the total dose of herbicide acquired throughout the feeding period before defecation (around 144 h of age) [32, 33]. Each larva received a total dose of GLY of 137.5, 275 and 550 ng a.e. per treatment. It is worth pointing out that the EFSA proposes that pesticide effects on beehives should be tested by using exposures with an average dose of 9500 ng a.e. per larva over the 5‐day developmental period before sealing [58]. This is almost 20 times greater than the highest doses we used. Detrimental effects on development were detected with GLY levels below 20.49 mg a.e. kg^-1^ in our study (estimated for ten 5-day-old larvae exposed to 1.25 mg a.e. L^-1^). In semi-field conditions, a previous study reported concentrations from 1.23 to 19.5 mg a.e. kg^-1^ in brood samples [35], without conspicuous developmental or survival effects. Nevertheless, we found a 21% reduction in weight of *in vitro* reared larvae with respect to larvae reared in-hive, which is evidence of malnutrition (S2 Fig). Larvae reared *in vitro* also showed less variability in weight at similar head diameter than larvae reared *in-hive*, due to both homogenised nutrition and constant food volume. Furthermore, honey bee larvae exposed to GLY *in vitro* showed symptoms of stress which would be compensated in hive by the care of nurse bees which promotes an allostatic load in the colony [5]. Hence, the malnutrition state and the absence of social immunity of larvae reared *in vitro* cannot be ignored.

In previous studies, larvae displayed different tolerance to stressors among colonies both in in-hive [59] and *in vitro* [38] contexts. In our study, results indicate variation in the susceptibility to GLY among colonies. *In vitro* rearing minimizes environmental stressors and homogenizes rearing conditions thus excluding other in-hive stressors which concurrently decompensate homeostasis. Variability of gene expression depending on the rearing context is also shown in our study, which could be a consequence of differences in the composition of the diet between in-hive and *in vitro* rearing. Furthermore, the absence of social immunity in *in vitro* rearing enhanced individual defences (S3 Fig).

The *in vitro* procedure does not mimic the pathway of exposure to agrochemicals under in-hive conditions in which the frequency and amount of food offered vary according to larval demand and supply provided by nurse bees [60, 61]. Therefore, *in vitro* exposure acts as a worst-case scenario with a predominant individual immunity. However, this procedure enables us to assess the specific responsiveness to GLY. Hence, inter-individual and inter-colony variance in susceptibility to stressors for the *in vitro* procedure becomes important. Furthermore, we can only assess the toxicity of the herbicide at the individual level.

### Outlook for toxicity assessment of GLY in honey bees

Nowadays agroecosystems are exposed to increasing amounts of GLY as a consequence of non-rational applications and resistant weeds that diminish the effectiveness of the herbicide [62]. Field monitoring and laboratory controlled assessments of GLY toxicity are still essential. The integration of information obtained from both procedures would be desirable to provide a better understanding of agrochemical impact on beehives exposed under realistic field conditions [63, 64]. Even if the *in vitro* procedure cannot be considered to completely reflect toxicity to larvae inside a hive, it can be considered as a reliable tool in a first step to determine subtle adverse effects. The impact of GLY-based herbicide formulations on the honey bee and the interaction with multiple factors such as pathogens, other pesticides or adverse environmental conditions, are unknown. However, our results suggest that the exposure to the active ingredient of these herbicides could affect brood development with unpredictable long-term consequences at the colony level. Assessments that do not take into account the variation of susceptibility among beehives may well incorrectly quantify adverse effects.

## Materials and methods

### Study site and animals

Experiments were performed from January to March during the summer season of the southern hemisphere. Larvae were sampled from six disease-free colonies (A-C in 2014 and D-F in 2015) of western honey bees (*Apis mellifera ligustica* Spinola) and reared *in vitro* (see below). Colonies were purchased from a commercial queen producer in November of each year and housed in new Langstroth hives at the experimental apiary of the University of Buenos Aires, Buenos Aires, Argentina (34° 32’ S, 58° 26’ W). The new six queens were not genetically related (different parents; i.e., inter-colony genetic diversity) and they were naturally inseminated by multiple mates during free flights in the field (i.e., intra-colony genetic diversity).

### *In vitro* rearing

We introduced an empty frame into the source colony (A-F) and monitored it for 8 hours until the queen had laid enough eggs. Three days later we withdrew the brood frame and carried it to a room suitable for grafting. We grafted around 120 first stadium larvae (0–8 hour old post-hatching) from the brood frame to plastic cups and placed them in Petri dishes. This amount of larvae represented around 10% of the cohort (eggs laid in one day by the queen) and up to 1% of the colony. To avoid variability in grafting effect the same researcher carried out this procedure. Larvae were reared inside an incubator with constant temperature and relative humidity (34.5°C and 95%, respectively) for five days [44]. In order to prevent bacterial or fungal contamination and subsequent infection, we maintained sterile conditions and removed dead ones daily [44]. To standardize larval food administration, we provided 160 μl of food spread in six aliquots of increasing volume to each larva during the six days of feeding period: 10 μl during grafting, 10 μl at 24 h, 20 μl at 48 h, 30 μl at 72 h, 40 μl at 96 h and 50 μl at 120 h [65]. We used a previously established diet: 6% D-glucose, 6% D-fructose and 50% commercial royal jelly [66, 67]. We performed one grafting session for each source colony per treatment every 2–3 days in order to exclude seasonal differences.

### Exposure to GLY

To evaluate the effect of exposure to GLY (analytical standard, purity of 99.2%) in larva reared *in vitro*, we assume the worst-case exposure scenario. We do not know the concentration of GLY in brood food during field exposure but GLY is actually ingested by larvae [36]. There is no biochemical procedure yet to measure accurately traces of GLY in royal jelly but other pesticides have been detected [22]. Consequently, we have chosen a chronic exposure to GLY considering environmental concentrations measured in natural and agricultural landscapes when recommended or excessive applications of the herbicide were used [23–25]. We defined four treatments: control group (food without herbicide), 1.25, 2.5 and 5 mg a.e. of GLY per litre of food. To prepare the food mixture for each concentration, we diluted a stock solution of 100 mg a.e. L^-1^ which we renewed once a week due to slight photodegradation of GLY [11]. Finally, we also measured the pH of the food mixture of each treatment during the experiment.

### Larval development

Throughout the growth/feeding period, four moults allow a honey bee larva to increase in size, which determines five stadia [32]. A moult normally occurs around every 24 hours up to the 4-day post-hatching (96 h). Each stadium can be identified daily by the head diameter of the larva and its morphology [32–34] (S1 Fig). When a larva has a smaller size or different characteristics from the stadium it is expected to be in, it was classified as delayed.

### Survival

Larvae can be classified as dead when their colour changes to brownish and they develop oedema, remain immobile and/or do not react to the contact of a paintbrush [44, 65]. We took note of their status daily.

### Food ingestion and growth

At the end of the feeding period (fifth stadium), larvae eat all offered food in both rearing contexts. Furthermore, inside the hive and prior to pupation, cells are sealed by nursing bees at around 120 hours after hatching. Hence, we sampled and weighed 5-day old larvae with complete food intake (110 μl of food ingested) from three colonies (D-F) to compare growth among the different GLY concentrations (10 larvae per concentration and colony). We used an electronic balance (Mettler Toledo AG285, ±0.1 mg) and measured the diameter of the head with a stereomicroscope (Leica MZ8) for a morphometric identification of the instar. As there are environmental, nutritional and social differences with in-hive rearing, we sampled larvae from the same cohort to compare nutritional baseline between contexts (S2 Fig). For in-hive rearing, we sampled in the three colonies ten 5-day old larvae from sealed cells prior to spinning (i.e. larvae remained at the bottom of the cell before changes its position).

### Dissection of the gut

We analysed the expression profile of some genes in the gut as a parameter to assess brood defences and health (Table 2). For this, we sampled 5-day old larvae in the fifth stadium from three colonies (D-F, in both rearing contexts as above section) with complete food intake (same dosage of GLY and nutritional status, S1 Table). We dissected them under a stereomicroscope and pooled 10 guts for each rearing context and each GLY treatment per colony. Pooled guts were immediately frozen in liquid nitrogen and stored at -80°C until RNA extraction.

### RNA extraction

We homogenized larval gut samples using a pestle and mortar in the presence of liquid nitrogen. Following the manufacturer’s instructions, polyadenylated RNA was extracted using the mRNA Isolation Kit (Roche Molecular Biochemicals). We quantified the concentration of mRNA using the Qubit fluorometer (Invitrogen).

### RT-PCR procedure

We estimated transcript accumulation of all genes through a semi-quantitative procedure with RT-PCR. cDNA was synthesized with 50 ng of mRNA per sample by means of the Revert AidTM M-Mul V Reverse transcriptase system (Fermentas International Inc.). PCRs were performed using GoTaq DNA polymerase (Promega). PCR specific primers for all analysed genes were designed for conserved nucleotide sequences (Table 2). Annealing conditions for each primer pair were optimized empirically to determine the linear range of amplification (S9 Table). *Actin* was used as an endogenous control to normalize the amount of starting template.

### Gene expression analysis

We separated RT-PCR products of the target genes and the endogenous control in 1.5% agarose gels (S4 Fig), stained with GelRed Nucleic Acid Stain (Biotium) and visualized by the UVP Doc-It LS Image Acquisition Software. We measured the intensity of the bands and compared them against a standard molecular marker loaded on the same gel (100-bp DNA ladder, Invitrogen). To analyse the response of each gene in exposure to glyphosate, we relativized their expression levels to their control baseline level.

### Statistics

We performed data analysis and graphics in R (for details see S1 Appendix). Survival and developmental data were analyzed with Accelerated failure-time models (ATF). Weighing data were analyzed with generalized linear models (GLM). Because of head diameter reached non-normality errors with different distributions, we performed a Kruskal-Wallis test. Hierarchical cluster analysis was performed with multiscale bootstrap resampling p-values to classify the genetic responsiveness for clustering in each edge. The alpha level was set at 0.05 and p-value corrected for multiple *post hoc* comparisons with Bonferroni procedure.

## Supporting information

S1 Appendix Statistical procedure and R programming. (PDF)

S1 Table Exposure conditions to glyphosate for the *in vitro* assessment. (PDF)

S2 Table Simple effects reported in ATF models with significant interaction. Multiple *post hoc* comparison of survival curves ([GLY] × colony term, χ^2^ (15) = 211.29, P < 0.001) and successful moulting curves ([GLY] × colony term, χ^2^ (15) = 207.24, P < 0.001) among treatments in each colony. Statistics of Log-rank tests (d.f. = 1) to compare a pair of GLY concentrations and p-value corrected with Bonferroni procedure (significant differences in bold).
(PDF)

S3 Table Simple effects for larvae reared *in vitro* without GLY reported in ATF models with significant interaction. Multiple *post hoc* comparison of control survival curves ([GLY] × colony term, χ^2^ (15) = 211.29, P < 0.001) and control successful moulting curves ([GLY] × colony term, χ^2^ (15) = 207.24, P < 0.001) among colonies. Statistics of Log-rank tests (d.f. = 1) to compare a pair of colonies below grey diagonal and p-value corrected with Bonferroni procedure above grey diagonal (significant differences in bold).
(PDF)

S4 Table Multiple post hoc comparison of head diameter among treatments. Statistics of Nemenyi test (d.f. = (15, 135)) to compare a pair of GLY concentrations or rearing contexts in each colony. P-value was corrected with Bonferroni procedure.
(PDF)

S5 Table Multiple post hoc comparison of head diameter among colonies. Statistics of Nemenyi test (d.f. = (15, 135)) to compare a pair of colonies in each rearing context. P-value was corrected with Bonferroni procedure.
(PDF)

S6 Table Simple effects reported in GLM model with significant interaction. Multiple *post hoc* comparison of weight among groups ([GLY] × colony term, F(6,108) = 16.33, P < 0.001, N = 120). Statistics of Tukey test to compare a pair of GLY concentrations in each colony. P-value was corrected with Bonferroni procedure (significant differences in bold).
(PDF)

S7 Table Simple effects reported in GLM model with significant interaction. Multiple *post hoc* comparison of weight among groups ([GLY] × colony term, F(6,108) = 16.33, P < 0.001, N = 120). Statistics of Tukey test to compare a pair of colonies in each GLY concentration. P-value was corrected with Bonferroni procedure (significant differences in bold).
(PDF)

S8 Table Changes in mean pH of food offered during the *in vitro* assessment. Five replicates per treatment have been measured daily throughout 5 days at incubator (34.5°C and 95% RH). GLMM followed by Tukey test to compare a pair of GLY concentrations. Treatments with different letters have significantly different means.
(PDF)

S9 Table Procedure conditions for each primer pair in the RT-PCR were optimized empirically to determine the linear range of amplification. (PDF)

S10 Table Comparison of gene expression levels assessed between rearing contexts. Statistics of Mann-Whitney *U* test to compare a pair of genes in each rearing context (in-hive or *in vitro*).
(PDF)

S1 Fig Larval development during the growth period. Day by day photographic sequence of the expected development. Growth and feeding period corresponds to the first 144 hours after hatching. A) 0–17 h: First stadium (I) larva (circled in red). The instar (1.5 mm) has a translucent cuticle and a head that is hard to observe with the naked eye. B) 17–36 h: Second stadium (II) larva. The instar (2 mm) has a visible head but with very small jaws and an opaque whitish cuticle. C) 36–57 h: Third stadium (III) larva. The instar (3 mm) has a robust appearance and a shiny whitish cuticle. D) 57–85 h: Fourth stadium (IV) larva. The instar (4 mm) has a head of large diameter. E) 85–115 h: Fifth stadium (V) larva. The early fifth instar (6 mm) has conspicuous jaws and shows a large increase in body mass in relation to the head. F) 115–160 h: The late fifth instar (8 mm) continues gorging in both contexts but inside the sealed cell in in-hive rearing.
(PDF)

S2 Fig Effect of rearing context on growth. Larvae without adverse symptoms in larval development were sampled at 5-day of age from three colonies (D, E and F) in both rearing contexts (in-hive and *in vitro*). We measured in each larva their (a) head diameter (mm) and (b) weight (mg). The number of larvae measured was 10 for each rearing context per colony. Kruskal-Wallis test (head diameter ~ treatment. Treatment as a combination of rearing context and colony) was carried out to analyse morphometric data to compare among groups (χ^2^ (5) = 16.48, P = 0.005). GLS was carried out to analyse weight data to compare among groups. (GLS model: weight ~ rearing context + colony + rearing context × colony. Fixed factors: LR (2) = 57.4, P < 0.001, N = 60. Rearing context × colony term, LR (1) = 0.45, P = 0.502. Rearing context term for variance structure: LR (2) = 54.82, P < 0.001). Groups with asterisks have significantly different means.
(PDF)

S3 Fig Effects of rearing context on gene expression within the epithelium gut. Measurement of the mean expression level of 16 genes has been performed in guts of 5-day-old dissected larvae sampled from three colonies (D, E and F) in both rearing contexts (in-hive or *in vitro*). A pool of 10 guts for each colony and context has been assessed (6 samples). *Actin* expression level has been used to normalize the expression level of every gene. Bars indicate means ± s.e.m. Mann-Whitney *U* test to compare between contexts for each gene (no significant differences, S10 Table).
(PDF)

S4 Fig Gel electrophoresis of RT-PCR products of the target genes in each larval gut sample. Pool samples of 10 guts of 5-day-old larvae (reared in-hive or *in vitro*) sampled from three colonies (D, E and F) exposed to different concentrations of glyphosate (1.25–5.0 mg. of GLY per litre of food). One agarose gel was performed for each gene on all samples.
(PDF)

S1 Dataset Row data of all measurements in the GLY assessment. (XLSX)
