# Supplementary material for: Glyphosate affects the larval development of honey bees depending on the susceptibility of colonies
Source: PLoS One. 2018 Oct 9;13(10):e0205074. doi: 10.1371/journal.pone.0205074 (PMC6177133; doi:10.1371/journal.pone.0205074)
Supplement: S1 Appendix — (PDF) [file pone.0205074.s001.pdf]

Glyphosate affects the larval development of honey bees depending on the susceptibility of colonies

Diego E. Vázquez[1,2], Natalia Ilina[3,4], Eduardo A. Pagano[3,4], Jorge A. Zavala[3,4] and Walter M. Farina[1,2,*]

[1] *Universidad de Buenos Aires, Facultad de Ciencias Exactas y Naturales, Departamento de Biodiversidad y Biología Experimental, Laboratorio de Insectos Sociales, Buenos Aires, Argentina.*

[2] *CONICET-Universidad de Buenos Aires, Instituto de Fisiología, Biología Molecular y Neurociencias (IFIBYNE), Buenos Aires, Argentina.*

[3] *Universidad de Buenos Aires, Facultad de Agronomía, Cátedra de Bioquímica, Buenos Aires, Argentina.*

[4] *CONICET-Universidad de Buenos Aires, Instituto de Investigaciones en Biociencias Agrícolas y Ambientales, (INBA), Buenos Aires, Argentina.*

[*]Corresponding author

E-mail: walter@fbmc.fcen.uba.ar (WMF)

Materials and Methods

Statistical procedure and R programming. Data analysis and graphics were performed in R version 3.3.3 [1, 2]. Survival and developmental data were analyzed with Accelerated failure-time models (ATF) using the survival package because we have censoring and age-specific hazard [3, 4]. These failure-time data was fitted to a model with Student *t* error structure and interaction between the fixed factors (GLY concentration and source colony) using the survreg function of the survival package. Post-hoc pairwise comparisons for each colony or among baselines were performed with the log-rank test using the survdiff function of the same package. Weighing data were analyzed with generalized linear models (GLM) to compare among *in vitro* groups using glm function. Meanwhile, weighing data from different rearing contexts were analyzed with generalized least square models (GLS) for modeling variance using gls function and varFunc objects of the nlme package [5]. In both cases, data were fitted to a model with Gaussian error structure and interaction between the fixed factors (source colony and treatment, ie. GLY concentration or rearing context). Changes in mean pH of contaminated food were analyzed with generalized linear mixed models (GLMM) with time and replicate as random factors while data was fitted to a model with Gaussian error structure. GLMM has been used to model variance due to repeated measurements using the lme function of nlme package [5]. Post-hoc pairwise comparisons for all combinations in weighing data and pH data were performed with Tukey test using the glht function of the multcomp package [6]. The selection of the most parsimonious model for ATF, GLM, GLS and GLMM was carried out assessing the relative importance of each factor with a stepwise subtraction method using anova function (approach with hypothesis testing based on comparing nested models) [7, 8]. Because of head diameter reached non-normality errors with different distributions, we performed a Kruskal-Wallis test (kruskal.test function) for comparison among treatments (factor resulting from the combination of source colonies with GLY concentration or rearing context). Post-hoc pairwise comparisons for all combinations of morphometric data were performed with Nemenyi test using the posthoc.kruskal.nemenyi.test function of the PMCMR package [9]. Genetic data were analyzed with Mann-Whitney *U* test (wilcox.test function) for comparison between contexts for each gene. Besides, hierarchical cluster analysis (pvclust function) was performed with the pvclust package to classify the genetic

responsiveness [10]. For all tests, we report the statistic with its degrees of freedom and p-value when the minimal adequate model was contrasted with the null model. The alpha level was set at 0.05 and p-value corrected for multiple *post hoc* comparisons with Bonferroni procedure (p-value' = p-value*k, k = number of comparisons).

References

1. R Core Team (2017). R: A language and environment for statistical computing. R Foundation for Statistical Computing, Vienna, Austria. URL: https://www.R-project.org/.
2. H. Wickham (2009). ggplot2: Elegant Graphics for Data Analysis. Springer-Verlag New York.
3. Therneau T.M. (2015). A Package for Survival Analysis in S. version 2.38, URL: https://CRAN.R-project.org/package=survival.
4. Therneau T.M. and Grambsch P.M. (2000). Modeling Survival Data Extending the Cox Model. Springer, New York. ISBN 0-387-98784-3.
5. Pinheiro J., Bates D., DebRoy S., Sarkar D. and R Core Team (2017). nlme: Linear and Nonlinear Mixed Effects Models. R package version 3.1-131, URL: https://CRAN.R-project.org/package=nlme.
6. Hothorn T., Bretz F. and Westfall P. (2008). Simultaneous Inference in General Parametric Models. Biometrical Journal 50(3), 346--363.
7. Zuur, A. F., Ieno, E. N., Walker, N. J., Saveliev, A. A., and Smith, G. M. (2009). Mixed effects models and extensions in ecology with R. Gail M, Krickeberg K, Samet JM, Tsiatis A, Wong W, editors. New York, NY: Spring Science and Business Media.
8. Crawley, M.J. (2012). The R book. Imperial College London at Silwood Park. UK, 527-528.
9. Pohlert T. (2014). The Pairwise Multiple Comparison of Mean Ranks Package (PMCMR). R package, URL: http://CRAN.R-project.org/package=PMCMR.
10. Suzuki R. and Shimodaira H. (2015). pvclust: Hierarchical Clustering with P-Values via Multiscale Bootstrap Resampling. R package version 2.0-0. https://CRAN.R-project.org/package=pvclust.
