# Supplementary material for: Glyphosate affects the larval development of honey bees depending on the susceptibility of colonies
Source: PLoS One. 2018 Oct 9;13(10):e0205074. doi: 10.1371/journal.pone.0205074 (PMC6177133; doi:10.1371/journal.pone.0205074)
Supplement: S1 Table — (PDF) [file pone.0205074.s002.pdf]

S1 Table. Exposure conditions to glyphosate for the *in vitro* assessment.

Exposure time (h)	Offered food (µL)		Cumulative dose (ng per larva) of glyphosate			
	Daily volume	Cumulative volume	Exposure concentration (mg L^{-1})			
			0.0	1.25	2.5	5.0
24	10	10	0	12.5	25	50
48	10	20	0	25	50	100
72	20	40	0	50	100	200
96	30	70	0	87.5	175	350
120	40	110	0	137.5	275	550
144	50	160	0	200	400	800
