# Supplementary material for: Glyphosate affects the larval development of honey bees depending on the susceptibility of colonies
Source: PLoS One. 2018 Oct 9;13(10):e0205074. doi: 10.1371/journal.pone.0205074 (PMC6177133; doi:10.1371/journal.pone.0205074)
Supplement: S2 Table — Multiple post hoc comparison of survival curves ([GLY] × colony term, χ2 (15) = 211.29, P < 0.001) and successful moulting curves ([GLY] × colony term, χ2 (15) = 207.24, P < 0.001) among treatments in each colony. Statistics of Log-rank tests (d.f. = 1) to compare a pair of GLY concentrations and p-value corrected with Bonferroni procedure (significant differences in bold). (PDF) [file pone.0205074.s003.pdf]

S2 Table. Simple effects reported in ATF models with significant interaction. Multiple *post hoc* comparison of survival curves ([GLY] × colony term, χ^2 (15) = 211.29, P < 0.001) and successful moulting curves ([GLY] × colony term, χ^2 (15) = 207.24, P < 0.001) among treatments in each colony. Statistics of Log-rank tests (d.f. = 1) to compare a pair of GLY concentrations and p-value corrected with Bonferroni procedure (significant differences in bold).

	GLY concentration (mg L^{-1})	Colony											
		A		B		C		D		E		F	
Effects	pairwise comparison	χ^2	P-value	χ^2	P-value	χ^2	P-value	χ^2	P-value	χ^2	P-value	χ^2	P-value
Survival	0 vs 1.25	0.3	1	0.1	1	**14.8**	**0.004**	7.5	0.22	4.1	1	0.7	1
	0 vs 2.5	0.9	1	6.6	0.378	**19.8**	**<0.001**	7.2	0.268	**15.5**	**0.003**	**10.9**	**0.035**
	0 vs 5	**60.7**	**<0.001**	0.9	1	**14.8**	**0.004**	0.5	1	10	0.057	0.6	1
	1.25 vs 2.5	0.2	1	5.2	0.832	**56.6**	**<0.001**	0	1	4.5	1	7.4	0.231
	1.25 vs 5	**66.7**	**<0.001**	0.4	1	0	1	**11.6**	**0.023**	1.7	1	2.8	1
	2.5 vs 5	**89.9**	**<0.001**	2.7	1	**61**	**<0.001**	**11.3**	**0.028**	0.7	1	**17.9**	**0.03**
Succesful moulting	0 vs 1.25	0.3	1	6.2	0.45	**102**	**<0.001**	**77.2**	**<0.001**	0.1	1	1.4	1
	0 vs 2.5	**10.9**	**0.035**	1.4	1	**18.1**	**<0.001**	**16.8**	**<0.001**	6.7	0.354	**17.4**	**<0.001**
	0 vs 5	8.6	0.118	4.6	1	**53.1**	**<0.001**	**18.5**	**<0.001**	9.8	0.064	**14.6**	**0.005**
	1.25 vs 2.5	2.1	1	2.5	1	**14.1**	**0.006**	**27.6**	**<0.001**	6.3	0.446	8.5	0.125
	1.25 vs 5	3.9	1	1.5	1	4.3	1	**32.4**	**<0.001**	9.5	0.074	**23.6**	**<0.001**
	2.5 vs 5	0.3	1	0.4	1	6.5	0.392	0	1	**32**	**<0.001**	**62.6**	**<0.001**
