# Supplementary material for: Glyphosate affects the larval development of honey bees depending on the susceptibility of colonies
Source: PLoS One. 2018 Oct 9;13(10):e0205074. doi: 10.1371/journal.pone.0205074 (PMC6177133; doi:10.1371/journal.pone.0205074)
Supplement: S3 Table — Multiple post hoc comparison of control survival curves ([GLY] × colony term, χ2 (15) = 211.29, P < 0.001) and control successful moulting curves ([GLY] × colony term, χ2 (15) = 207.24, P < 0.001) among colonies. Statistics of Log-rank tests (d.f. = 1) to compare a pair of colonies below grey diagonal and p-value corrected with Bonferroni procedure above grey diagonal (significant differences in bold). (PDF) [file pone.0205074.s004.pdf]

S3 Table. Simple effects for larvae reared *in vitro* without GLY reported in ATF models with significant interaction. Multiple *post hoc* comparison of control survival curves ([GLY] × colony term, χ^2 (15) = 211.29, P < 0.001) and control successful moulting curves ([GLY] × colony term, χ^2 (15) = 207.24, P < 0.001) among colonies. Statistics of Log-rank tests (d.f. = 1) to compare a pair of colonies below grey diagonal and p-value corrected with Bonferroni procedure above grey diagonal (significant differences in bold).

		Pairwise comparison between colonies (*in vitro* rearing without GLY)					
Effects	Colony	A	B	C	D	E	F
Survival	A		1	**0.001**	0.3	0.3	1
	B	2.3		0.176	**0.002**	1	0.495
	C	**15.3**	6.3		**<0.001**	1	**<0.001**
	D	5.04	**15.1**	**39.8**		**<0.001**	1
	E	5.2	0.6	2.5	**20.2**		0.069
	F	0.4	4.5	**20.5**	2.9	8	
Successful moulting	A		1	0.114	**0.016**	1	1
	B	0		0.122	**0.017**	1	1
	C	7.1	7		1	0.315	0.058
	D	**10.7**	**10.7**	0.4		0.085	**0.009**
	E	0.2	0.1	3.5	7.6		1
	F	0	0.1	8.4	**11.8**	0.4	
