# Supplementary material for: Glyphosate affects the larval development of honey bees depending on the susceptibility of colonies
Source: PLoS One. 2018 Oct 9;13(10):e0205074. doi: 10.1371/journal.pone.0205074 (PMC6177133; doi:10.1371/journal.pone.0205074)
Supplement: S4 Table — Statistics of Nemenyi test (d.f. = (15, 135)) to compare a pair of GLY concentrations or rearing contexts in each colony. P-value was corrected with Bonferroni procedure. (PDF) [file pone.0205074.s005.pdf]

S4 Table. Multiple post hoc comparison of head diameter among treatments. Statistics of Nemenyi test (d.f. = (15, 135)) to compare a pair of GLY concentrations or rearing contexts in each colony. P-value was corrected with Bonferroni procedure.

	Colony	D		E		F	
	pairwise comparison	Statistic q	P-value	Statistic q	P-value	Statistic q	P-value
GLY concentration (mg/L)	0 vs 1.25	0	1	0.59	1	0.59	1
	0 vs 2.5	2.77	1	0.59	1	1.17	1
	0 vs 5	0.59	1	1.17	1	0.59	1
	1.25 vs 2.5	2.77	1	2.42	1	0.59	1
	1.25 vs 5	0.59	1	0.59	1	0	1
	2.5 vs 5	2.18	1	3.01	1	0.59	1
	in-hive vs *in vitro*	4.3	1	2.94	1	0.59	1
