# Supplementary material for: Glyphosate affects the larval development of honey bees depending on the susceptibility of colonies
Source: PLoS One. 2018 Oct 9;13(10):e0205074. doi: 10.1371/journal.pone.0205074 (PMC6177133; doi:10.1371/journal.pone.0205074)
Supplement: S5 Table — Statistics of Nemenyi test (d.f. = (15, 135)) to compare a pair of colonies in each rearing context. P-value was corrected with Bonferroni procedure. (PDF) [file pone.0205074.s006.pdf]

S5 Table. Multiple post hoc comparison of head diameter among colonies. Statistics of Nemenyi test (d.f. = (15, 135)) to compare a pair of colonies in each rearing context. P-value was corrected with Bonferroni procedure.

	Rearing context	in-hive		*in vitro*	
	pairwise comparison	Statistic q	P-value	Statistic q	P-value
Colony	D vs E	0.19	1	1.17	1
	D vs F	2.54	1	1.17	1
	E vs F	2.36	1	0	1
