# Supplementary material for: Glyphosate affects the larval development of honey bees depending on the susceptibility of colonies
Source: PLoS One. 2018 Oct 9;13(10):e0205074. doi: 10.1371/journal.pone.0205074 (PMC6177133; doi:10.1371/journal.pone.0205074)
Supplement: S6 Table — Multiple post hoc comparison of weight among groups ([GLY] × colony term, F(6,108) = 16.33, P < 0.001, N = 120). Statistics of Tukey test to compare a pair of GLY concentrations in each colony. P-value was corrected with Bonferroni procedure (significant differences in bold). (PDF) [file pone.0205074.s007.pdf]

S6 Table. Simple effects reported in GLM model with significant interaction. Multiple *post hoc* comparison of weight among groups ([GLY] × colony term, $F(6,108) = 16.33$, $P < 0.001$, $N = 120$). Statistics of Tukey test to compare a pair of GLY concentrations in each colony. P-value was corrected with Bonferroni procedure (significant differences in bold).

	Colony	D		E		F	
	pairwise comparison	Statistic Z	P-value	Statistic Z	P-value	Statistic Z	P-value
GLY concentration (mg L^{-1})	0 vs 1.25	-2.94	0.148	0.51	1	**-3.61**	**0.019**
	0 vs 2.5	**-9.82**	**<0.01**	1.05	0.999	-2.48	0.398
	0 vs 5	**-10.08**	**<0.01**	0.96	0.999	-1.78	0.88
	1.25 vs 2.5	**-6.65**	**<0.01**	0.5	1	1.67	0.924
	1.25 vs 5	**-7.11**	**<0.01**	0.42	1	2.22	0.596
	2.5 vs 5	-0.91	0.999	-0.07	1	0.72	1
