# Supplementary material for: Glyphosate affects the larval development of honey bees depending on the susceptibility of colonies
Source: PLoS One. 2018 Oct 9;13(10):e0205074. doi: 10.1371/journal.pone.0205074 (PMC6177133; doi:10.1371/journal.pone.0205074)
Supplement: S7 Table — Multiple post hoc comparison of weight among groups ([GLY] × colony term, F(6,108) = 16.33, P < 0.001, N = 120). Statistics of Tukey test to compare a pair of colonies in each GLY concentration. P-value was corrected with Bonferroni procedure (significant differences in bold). (PDF) [file pone.0205074.s008.pdf]

S7 Table. Simple effects reported in GLM model with significant interaction. Multiple *post hoc* comparison of weight among groups ([GLY] × colony term, $F(6,108) = 16.33$, $P < 0.001$, $N = 120$). Statistics of Tukey test to compare a pair of colonies in each GLY concentration. P-value was corrected with Bonferroni procedure (significant differences in bold).

		GLY concentration (mg L^{-1})							
		0		1.25		2.5		5	
	pairwise comparison	Statistic Z	P-value	Statistic Z	P-value	Statistic Z	P-value	Statistic Z	P-value
Colony	D vs E	**-9.41**	**<0.01**	**-6.7**	**<0.01**	-0.92	0.999	-0.03	1
	D vs F	**-5.69**	**<0.01**	**-6.62**	**<0.01**	0.64	1	2.14	0.662
	E vs F	**4.08**	**<0.01**	0.11	1	1.56	0.956	2.16	0.641
