# Supplementary material for: Glyphosate affects the larval development of honey bees depending on the susceptibility of colonies
Source: PLoS One. 2018 Oct 9;13(10):e0205074. doi: 10.1371/journal.pone.0205074 (PMC6177133; doi:10.1371/journal.pone.0205074)
Supplement: S8 Table — Five replicates per treatment have been measured daily throughout 5 days at incubator (34.5°C and 95% RH). GLMM followed by Tukey test to compare a pair of GLY concentrations. Treatments with different letters have significantly different means. (PDF) [file pone.0205074.s009.pdf]

S8 Table. Changes in mean pH of food offered during the *in vitro* assessment. Five replicates per treatment have been measured daily throughout 5 days at incubator (34.5 °C and 95% RH). GLMM followed by Tukey test to compare a pair of GLY concentrations. Treatments with different letters have significantly different means.

		GLY concentration (mg L^{-1})			
Incubation time (hs)	pH	0.0	1.25	2.5	5.0
0	mean	4.94	4.86	4.84	4.64
	SD	0.05	0.05	0.05	0.05
24	mean	4.86	4.82	4.76	4.64
	SD	0.05	0.08	0.05	0.05
48	mean	4.98	4.90	4.74	4.56
	SD	0.13	0.07	0.13	0.19
72	mean	4.92	4.82	4.80	4.70
	SD	0.28	0.16	0.16	0.12
96	mean	4.96	4.82	4.68	4.58
	SD	0.19	0.11	0.15	0.08
120	mean	4.72	4.88	4.84	4.72
	SD	0.16	0.08	0.05	0.15
mean per treatment		**4.90**[a]	**4.85**[ab]	**4.78**[b]	**4.64**[c]
SD per treatment		0.18	0.10	0.12	0.12
