# Supplementary material for: Glyphosate affects the larval development of honey bees depending on the susceptibility of colonies
Source: PLoS One. 2018 Oct 9;13(10):e0205074. doi: 10.1371/journal.pone.0205074 (PMC6177133; doi:10.1371/journal.pone.0205074)
Supplement: S9 Table — (PDF) [file pone.0205074.s010.pdf]

S9 Table. Procedure conditions for each primer pair in the RT-PCR were optimized empirically to determine the linear range of amplification.

Gene Target	Fragment size (pb)	Denaturing	Annealing	Extension	N° of cycles
CYP6AS2	206	50 " 95°C	45 " 54°C	1 ' 72°C	34
CYP6AS3	116	50 " 95°C	45 " 54°C	1 ' 72°C	34
CYP6AS4	252	50 " 95°C	45 " 54°C	1 ' 72°C	47
CYP6AS5	302	50 " 95°C	45 " 54°C	1 ' 72°C	34
CYP6BD1	218	50 " 95°C	1 ' 56°C	1 ' 72°C	42
CYP9Q3	364	50 " 95°C	45 " 54°C	1 ' 72°C	42
Esterase FE4-like	243	50 " 95°C	1 ' 53°C	1 ' 72°C	44
Carboxylesterase	271	50 " 95°C	1 ' 54°C	1 ' 72°C	38
GstD1	272	50 " 95°C	1 ' 54°C	1 ' 72°C	39
Abaecin	110	50 " 95°C	45 " 54°C	1 ' 72°C	42
hsp70	217	50 " 95°C	1 ' 53°C	1 ' 72°C	33
Hsc70-3	184	50 " 95°C	1 ' 53°C	1 ' 72°C	32
Cys-proteinase	267	50 " 95°C	1 ' 54°C	1 ' 72°C	38
Cathepsine L1	161	50 " 95°C	1 ' 54°C	1 ' 72°C	39
Alpha-glucosidase	181	50 " 95°C	1 ' 54°C	1 ' 72°C	38
Alpha-amylase	231	50 " 95°C	1 ' 54°C	1 ' 72°C	39
Actin	176	50 " 95°C	45 " 54°C	1 ' 72°C	32
