# Supplementary material for: Glyphosate affects the larval development of honey bees depending on the susceptibility of colonies
Source: PLoS One. 2018 Oct 9;13(10):e0205074. doi: 10.1371/journal.pone.0205074 (PMC6177133; doi:10.1371/journal.pone.0205074)
Supplement: S10 Table — Statistics of Mann-Whitney U test to compare a pair of genes in each rearing context (in-hive or in vitro). (PDF) [file pone.0205074.s011.pdf]

S10 Table. Comparison of gene expression levels assessed between rearing contexts. Statistics of Mann-Whitney *U* test to compare a pair of genes in each rearing context (in-hive or *in vitro*).

	in-hive vs *in vitro*		
	pairwise comparison	Statistic W	p-value
Locus	CYP6AS2	3	0.513
	CYP6AS3	3	0.513
	CYP6AS4	3	0.513
	CYP6AS5	1	0.127
	CYP6BD1	0	0.05
	CYP9Q3	4	0.827
	Esterase FE4-like	0	0.05
	Carboxylesterase	7	0.275
	GstD1	9	0.05
	Abaecin	3	0.513
	Hsp70	6	0.513
	Hsc70-30	7	0.275
	CYS-proteinase	8	0.127
	Cathepsin-L1	6	0.513
	Alpha-amylase	6	0.513
	AGLU2	7	0.275
