# Supplementary material for: Glyphosate affects the larval development of honey bees depending on the susceptibility of colonies
Source: PLoS One. 2018 Oct 9;13(10):e0205074. doi: 10.1371/journal.pone.0205074 (PMC6177133; doi:10.1371/journal.pone.0205074)
Supplement: S1 Fig — Day by day photographic sequence of the expected development. Growth and feeding period corresponds to the first 144 hours after hatching. A) 0–17 h: First stadium (I) larva (circled in red). The instar (1.5 mm) has a translucent cuticle and a head that is hard to observe with the naked eye. B) 17–36 h: Second stadium (II) larva. The instar (2 mm) has a visible head but with very small jaws and an opaque whitish cuticle. C) 36–57 h: Third stadium (III) larva. The instar (3 mm) has a robust appearance and a shiny whitish cuticle. D) 57–85 h: Fourth stadium (IV) larva. The instar (4 mm) has a head of large diameter. E) 85–115 h: Fifth stadium (V) larva. The early fifth instar (6 mm) has conspicuous jaws and shows a large increase in body mass in relation to the head. F) 115–160 h: The late fifth instar (8 mm) continues gorging in both contexts but inside the sealed cell in in-hive rearing. (PDF) [file pone.0205074.s012.pdf]

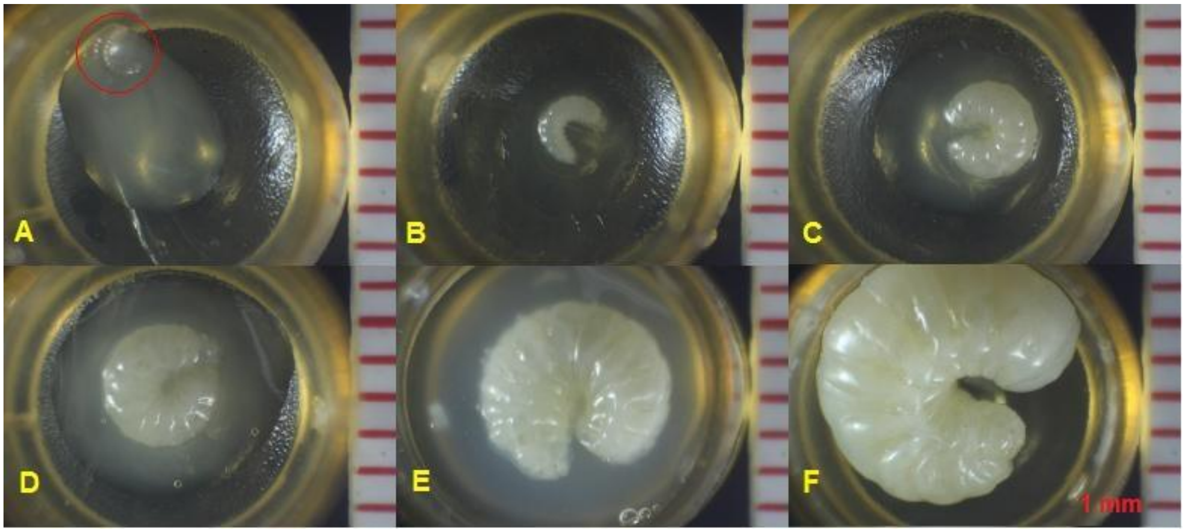

S1 Fig. Larval development during the growth period. Day by day photographic sequence of the expected development. Growth and feeding period corresponds to the first 144 hours after hatching. **A)** 0-17 h: First stadium (I) larva (circled in red). The instar (1.5 mm) has a translucent cuticle and a head that is hard to observe with the naked eye. **B)** 17-36 h: Second stadium (II) larva. The instar (2 mm) has a visible head but with very small jaws and an opaque whitish cuticle. **C)** 36-57 h: Third stadium (III) larva. The instar (3 mm) has a robust appearance and a shiny whitish cuticle. **D)** 57-85 h: Fourth stadium (IV) larva. The instar (4 mm) has a head of large diameter. **E)** 85-115 h: Fifth stadium (V) larva. The early fifth instar (6 mm) has conspicuous jaws and shows a large increase in body mass in relation to the head. **F)** 115-160 h: The late fifth instar (8 mm) continues gorging in both contexts but inside the sealed cell in in-hive rearing.
