# Supplementary material for: Glyphosate affects the larval development of honey bees depending on the susceptibility of colonies
Source: PLoS One. 2018 Oct 9;13(10):e0205074. doi: 10.1371/journal.pone.0205074 (PMC6177133; doi:10.1371/journal.pone.0205074)
Supplement: S2 Fig — Larvae without adverse symptoms in larval development were sampled at 5-day of age from three colonies (D, E and F) in both rearing contexts (in-hive and in vitro). We measured in each larva their (a) head diameter (mm) and (b) weight (mg). The number of larvae measured was 10 for each rearing context per colony. Kruskal-Wallis test (head diameter ~ treatment. Treatment as a combination of rearing context and colony) was carried out to analyse morphometric data to compare among groups (χ2 (5) = 16.48, P = 0.005). GLS was carried out to analyse weight data to compare among groups. (GLS model: weight ~ rearing context + colony + rearing context × colony. Fixed factors: LR (2) = 57.4, P < 0.001, N = 60. Rearing context × colony term, LR (1) = 0.45, P = 0.502. Rearing context term for variance structure: LR (2) = 54.82, P < 0.001). Groups with asterisks have significantly different means. (PDF) [file pone.0205074.s013.pdf]

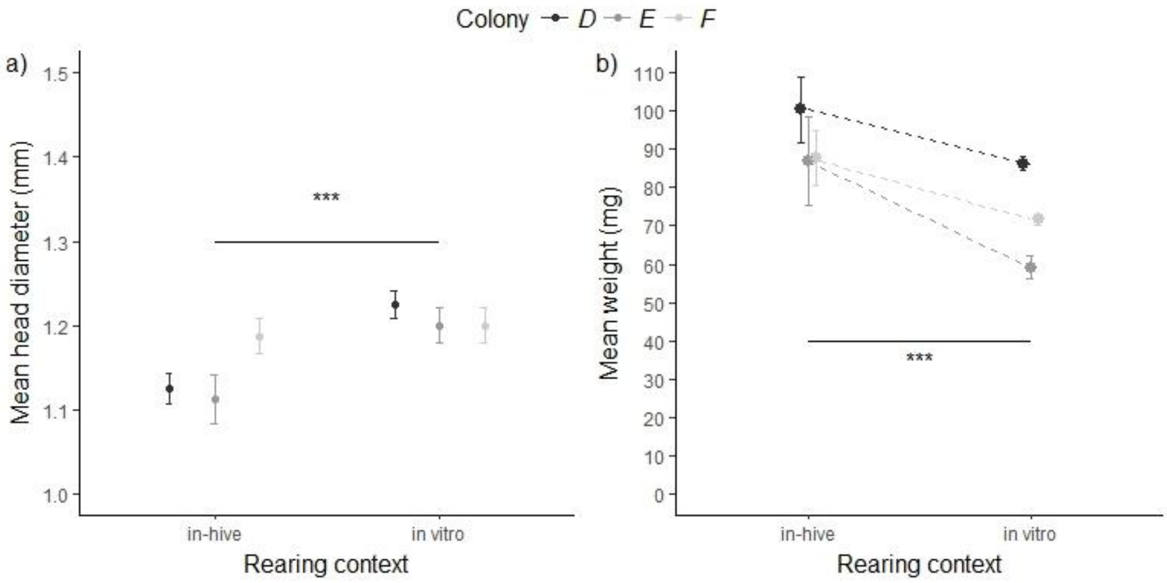

S2 Fig. Effect of rearing context on growth. Larvae without adverse symptoms in larval development were sampled at 5-day of age from three colonies (D, E and F) in both rearing contexts (in-hive and *in vitro*). We measured in each larva their **a)** head diameter (mm) and **b)** weight (mg). The number of larvae measured was 10 for each rearing context per colony. Kruskal-Wallis test (head diameter ~ treatment. Treatment as a combination of rearing context and colony) was carried out to analyse morphometric data to compare among groups (χ^2 (5) = 16.48, P = 0.005). GLS was carried out to analyse weight data to compare among groups. (GLS model: weight ~ rearing context + colony + rearing context × colony. Fixed factors: LR (2) = 57.4, P < 0.001, N = 60. Rearing context × colony term, LR (1) = 0.45, P = 0.502. Rearing context term for variance structure: LR (2) = 54.82, P < 0.001). Groups with asterisks have significantly different means.
