# Supplementary material for: Glyphosate affects the larval development of honey bees depending on the susceptibility of colonies
Source: PLoS One. 2018 Oct 9;13(10):e0205074. doi: 10.1371/journal.pone.0205074 (PMC6177133; doi:10.1371/journal.pone.0205074)
Supplement: S3 Fig — Measurement of the mean expression level of 16 genes has been performed in guts of 5-day-old dissected larvae sampled from three colonies (D, E and F) in both rearing contexts (in-hive or in vitro). A pool of 10 guts for each colony and context has been assessed (6 samples). Actin expression level has been used to normalize the expression level of every gene. Bars indicate means ± s.e.m. Mann-Whitney U test to compare between contexts for each gene (no significant differences, S10 Table). (PDF) [file pone.0205074.s014.pdf]

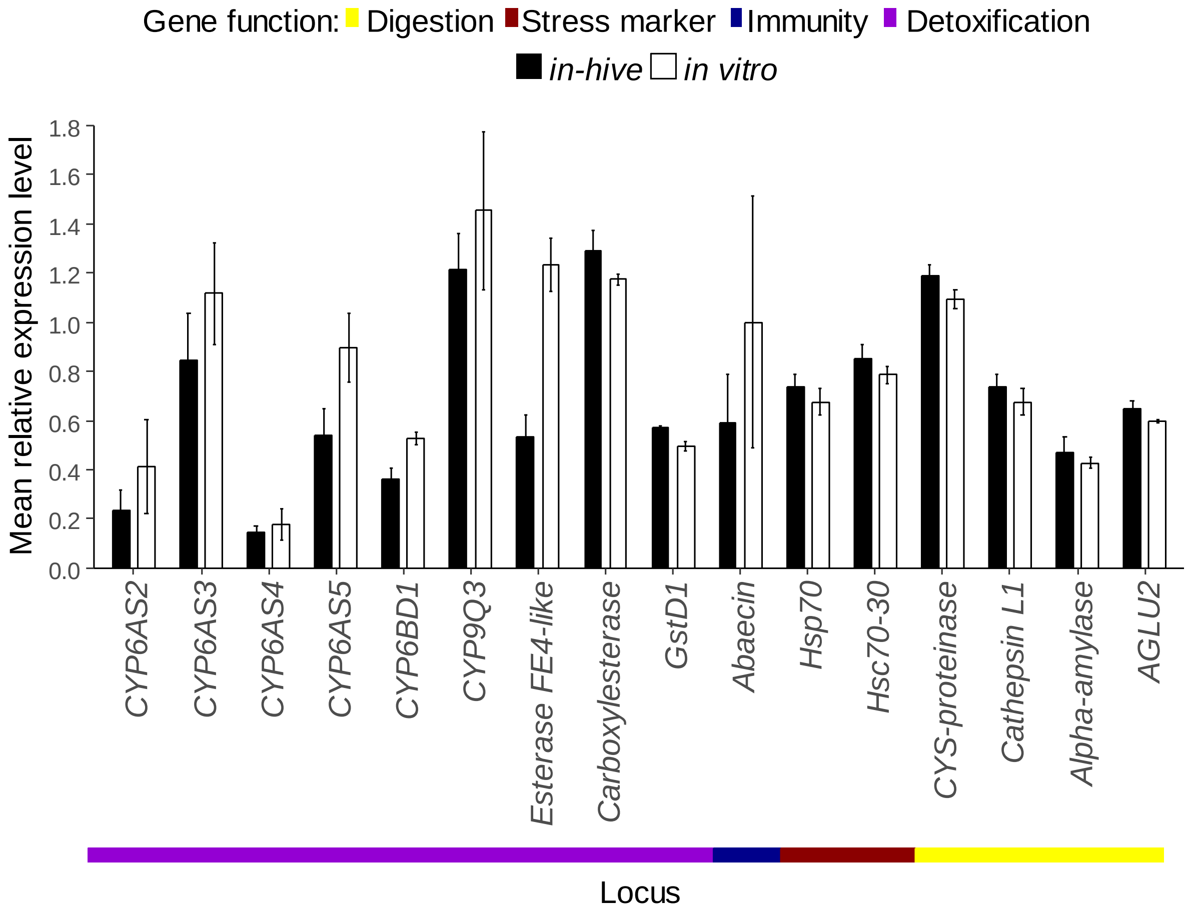

S3 Fig. Effects of rearing context on gene expression within the epithelium gut. Measurement of the mean expression level of 16 genes has been performed in guts of 5-day-old dissected larvae sampled from three colonies (D, E and F) in both rearing contexts (in-hive or *in vitro*). A pool of 10 guts for each colony and context has been assessed (6 samples). *Actin* expression level has been used to normalize the expression level of every gene. Bars indicate means ± s.e.m. Mann-Whitney *U* test to compare between contexts for each gene (no significant differences, S10 Table).
