# Supplementary material for: Glyphosate affects the larval development of honey bees depending on the susceptibility of colonies
Source: PLoS One. 2018 Oct 9;13(10):e0205074. doi: 10.1371/journal.pone.0205074 (PMC6177133; doi:10.1371/journal.pone.0205074)
Supplement: S4 Fig — Pool samples of 10 guts of 5-day-old larvae (reared in-hive or in vitro) sampled from three colonies (D, E and F) exposed to different concentrations of glyphosate (1.25–5.0 mg. of GLY per litre of food). One agarose gel was performed for each gene on all samples. (PDF) [file pone.0205074.s015.pdf]

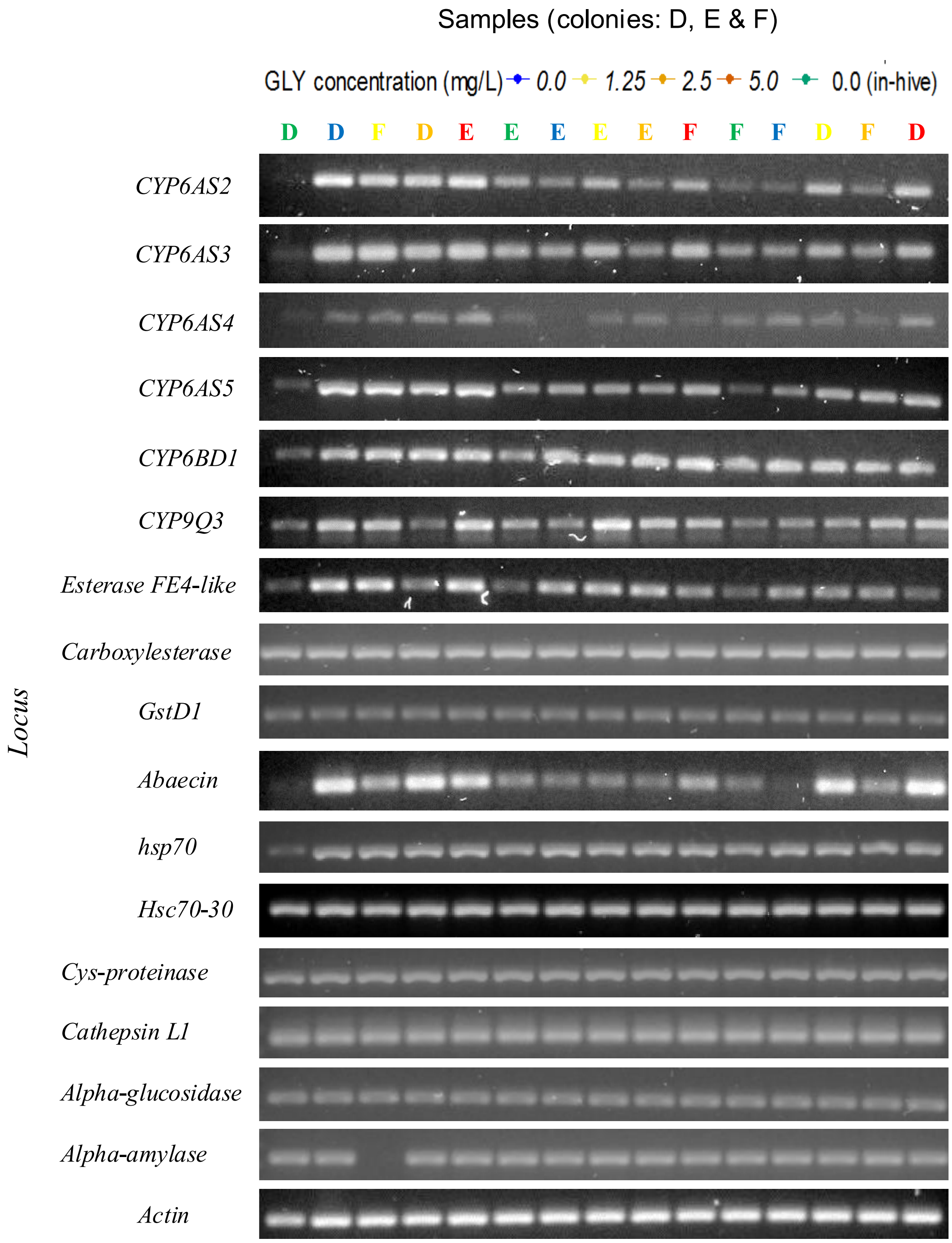

S4 Fig. Gel electrophoresis of RT-PCR products of the target genes in each larval gut sample. Pool samples of 10 guts of 5-day-old larvae (reared in-hive or *in vitro*) sampled from three colonies (D, E and F) exposed to different concentrations of glyphosate (1.25-5.0 mg. of GLY per litre of food). One agarose gel was performed for each gene on all samples.
